# Primary HSV-2 Infection in an Immunocompromised Patient Reveals High Diversity of Drug-Resistance Mutations in the Viral DNA Polymerase

**DOI:** 10.3390/v17070962

**Published:** 2025-07-09

**Authors:** Hanna Helena Schalkwijk, Sarah Gillemot, Emilie Frobert, Florence Morfin, Sophie Ducastelle, Anne Conrad, Pierre Fiten, Ghislain Opdenakker, Robert Snoeck, Graciela Andrei

**Affiliations:** 1 Molecular Structural and Translational Virology Research Group, Department of Microbiology, Immunology and Transplantation, Rega Institute for Medical Research, KU Leuven, 3000 Leuven, Belgiumrobert.snoeck@kuleuven.be (R.S.); 2 Virpath Unit, CIRI, Inserm U1111, CNRS, UMR5308, ENS Lyon, Université Claude Bernard Lyon 1 and Virology Unit, Institut des Agents Infectieux, Groupement Hospitalier Nord, Hospices Civils de Lyon, 69004 Lyon, France; 3 Hematology Clinical Unit, Groupement Hospitalier Sud, Hospices Civils de Lyon, 69495 Pierre-Bénite, France; 4 Infectious and Tropical Diseases Unit, Groupement Hospitalier Nord, Hospices Civils de Lyon, 69004 Lyon, France; 5 Laboratory of Immunobiology, Department of Microbiology, Immunology and Transplantation, Rega Institute for Medical Research, KU Leuven, 3000 Leuven, Belgium

**Keywords:** herpes simplex virus 2, drug resistance, viral heterogeneity, viral fitness

## Abstract

Herpes simplex virus 2 (HSV-2) remains a significant cause of morbidity and mortality in immunocompromised individuals, despite the availability of effective antivirals. Infections caused by drug-resistant isolates are an emerging concern among these patients. Understanding evolutionary aspects of HSV-2 resistance is crucial for designing improved therapeutic strategies. Here, we characterized 11 HSV-2 isolates recovered from various body sites of a single immunocompromised patient suffering from a primary HSV-2 infection unresponsive to acyclovir and foscarnet. The isolates were analyzed phenotypically and genotypically (Sanger sequencing of viral thymidine kinase and DNA polymerase genes). Viral clone isolations, deep sequencing, viral growth kinetics, and dual infection competition assays were performed retrospectively to assess viral heterogeneity and fitness. Sanger sequencing identified mixed populations of DNA polymerase mutant variants. Viral clones were plaque-purified and genotyped, revealing 17 DNA polymerase mutations (K533E, A606V, C625R, R628C, A724V, S725G, S729N, I731F, Q732R, M789T/K, Y823C, V842M, R847C, F923L, T934A, and R964H) associated with acyclovir and foscarnet resistance. Deep-sequencing of the DNA polymerase detected drug-resistant variants ranging between 1 and 95%, although the first two isolates had a wild-type DNA polymerase. Some mutants showed reduced fitness, evidenced by (i) the frequency of variants identified by deep-sequencing not correlating with the proportion of mutants found by plaque-purification, (ii) loss of the variants upon passaging in cell culture, or (iii) reduced frequencies in competition assays. This study reveals the rapid evolution of heterogeneous drug-resistant HSV-2 populations under antiviral therapy, highlighting the need for alternative treatment options and resistance surveillance, especially in severe infections.

## 1. Introduction

Herpes simplex virus 2 (HSV-2) is a human pathogen that causes chronic incurable infections worldwide. It has a global seroprevalence of 13.2% in the population aged 15–49 years and is the most common cause of genital ulcers in many countries [1,2]. Genital herpes is characterized by painful, vesicular, and ulcerative lesions. Following primary infection, HSV-2 establishes latency in sensory neurons, from which it periodically reactivates. Asymptomatic shedding plays a key role in transmission, with HSV-2 being shed (a)symptomatically at an average of 1 day per month [3]. The immune system plays a pivotal role in determining the severity of HSV-2 infections and the reactivation frequency [4]. Immunocompromised individuals and neonates are therefore at risk of developing severe HSV-2 infections that may have an atypical appearance with a risk of viral dissemination [5].

The nucleoside analog acyclovir and its prodrug valacyclovir are frequently used as treatment for primary and recurrent HSV-2 infections as well as chronic suppressive therapy to prevent recurrences [1,6]. While these drugs effectively reduce symptoms, they fail to prevent asymptomatic shedding, limiting their ability to reduce transmission risk [7,8]. Long-term treatment or prophylaxis can lead to the emergence of drug resistance, particularly in an immunocompromised host.

Most acyclovir-resistant mutations are found in the viral thymidine kinase (TK), the enzyme required for acyclovir activation. Mutations in the viral DNA polymerase (DP), the molecular target of acyclovir, are observed less frequently [9]. Alternative treatment options consist of other DP inhibitors, i.e., foscarnet, and cidofovir, which are both independent of TK activity. However, acyclovir-resistant mutations in the DP often confer cross-resistance to other DP inhibitors.

Here, we report a primary HSV-2 infection in a patient with medullary aplasia with a rapid onset of drug resistance due to highly heterogeneous viral populations comprising over 20 distinct drug-resistant variants.

## 2. Materials and Methods

### 2.1. Cells and Viruses

Human embryonic lung (HEL) fibroblasts were maintained in Dulbecco’s modified Eagle’s medium (DMEM, Thermo Fisher, Merelbeke, Belgium) supplemented with 8% fetal bovine serum (FBS). The HSV-2 reference strain G (ATCC VR-734) and clinical samples were grown on HEL fibroblasts in DMEM (2% FBS) to prepare virus culture stocks. Virus clones were isolated from the virus culture stock by serial dilution on HEL fibroblasts.

### 2.2. Compounds

The sources of compounds were adefovir (ADV) and cidofovir (CDV) [kindly provided by Gilead sciences, Foster City, CA, USA]; acyclovir (ACV) and foscarnet (phosphonoformic acid, PFA) [Merck, Hoeilaart, Belgium]; and ganciclovir (GCV, cymevene) [Roche, Basel, Switzerland].

### 2.3. Sanger Sequencing

DNA was extracted from the clinical samples and from the virus clones with the QIAamp DNA blood kit (Qiagen, Venlo, The Netherlands). The entire TK (*UL23*) and DP (*UL30*) genes were amplified in 3 and 9 overlapping amplicons, respectively, using M13 labeled primers (Table S1) with the FastStart High Fidelity PCR system (Roche). PCR products were purified (QIAquick PCR purification kit, Qiagen) and sequenced (BigDye Terminator v3.1, Thermo Fisher Scientific, Merelbeke, Belgium) on an ABI3730 sequencer (Applied Biosystems, Merelbeke, Belgium). Sequencing results were aligned to the HSV-2 G reference strain (GenBank accession number OM370995.1) in the SeqScape v2.7 software (Applied Biosystems).

### 2.4. Deep Sequencing

The frequency of mutations in the clinical samples was assessed by amplicon-based next-generation sequencing before and after five passages in cell culture. The partial DP gene (*UL30*, c.902-3223), encompassing the regions where drug-resistance mutations are located, was amplified with the Expand Long Template PCR system (Roche) using the following primers: DP forward 5′-CCACCCGGTTTATCCTGGACAAC-3′ and DP reverse 5′-CCTTGATGGACGGGACCTGC-3′. The amplicons were purified and prepared for sequencing on the Illumina Miseq v.2 as described previously [10]. Data analysis was performed with CLC Genomics Workbench using the HSV-2 G strain as a reference. The DNA polymerase sequences of the clinical samples have been submitted to the GenBank SRA database under BioProject PRJNA1219479.

### 2.5. Cytopathic Effect Reduction Assays

HEL fibroblasts were inoculated with the different virus culture stocks and virus clone stocks at an input of 100-fold the 50% cell culture infective dose (CCID_50_). The medium was replaced 2 h postinfection, after which serial dilutions of antiviral compounds were added. CPE was scored 72 h postinfection, and the concentration of drug that reduces virus CPE by 50% (EC_50_) was defined.

### 2.6. Growth Kinetics

Twenty-four-well plates with confluent monolayers of HEL fibroblasts were infected with 100 plaque forming units (PFUs) of the different virus stocks and incubated for 2 h, after which the medium was replaced. The plates were frozen on Days 1, 2, 3, 4, and 7 post infection at −80 °C, and virus titers (in PFU/mL) were determined upon thawing by serial dilution. GraphPad Prism software (version 10.3.1) was used for statistical analysis. A two-way ANOVA with Bonferroni correction was used to compare the replication capacities of mutant viruses to those of the wild type. A *p*-value < 0.05 was considered statistically significant.

### 2.7. Dual Infection Competition Assays

Dual-infection competition assays were performed as previously described [11]. Briefly, HEL fibroblasts grown in 24-well plates were infected with 100 PFUs/well at a 50:50 ratio of wild-type and mutant viruses or two mutant viruses and incubated in the presence of acyclovir (1 µg/mL), cidofovir (1 µg/mL), foscarnet (100 µg/mL), or no antiviral. Seven days postinfection, the frequency of each variant was determined by deep sequencing, as described above. Statistical analyses were performed in GraphPad Prism with the Brown–Forsythe and Welch analysis of variance (*p* ≤ 0.05), comparing the experiments under antiviral pressure to the untreated control experiment.

## 3. Results

### 3.1. Clinical History

A 30-year-old woman with medullary aplasia was hospitalized after a sudden onset of high fever (40 °C), painful swallowing, ulcerative necrotic angina, right cervical lymphadenopathy, sensitivity to pressure in the right hypochondrium, and disseminated mucocutaneous vesicular lesions mainly localized to the thorax, arms, and lower limbs. Herpes serology tested negative two months prior, despite a history of recurrent herpes labialis that benefited from valacyclovir treatment.

Upon hospitalization (Day +0), the patient presented pancytopenia, predominantly of platelets and leukocytes, low hemoglobin, and elevated CRP, creatinine, and transaminases. Skin, esophageal, oral, vaginal, and even plasma samples were HSV-2 positive. HSV-2 serology was positive for IgM (index of 3.4) but negative for IgG, confirming a primary HSV-2 infection. Acyclovir treatment was started (15 mg/kg/8 h), and antibiotic treatment with amikacin (initiated at the Emergency Department) and piperacillin/tazobactam was continued.

The patient revealed a vesicular crusty rash on the face, thorax, abdomen, anterior surface of the thighs, and anogenital area, accompanied with dysuria. Due to the painful and hypertensive appearance of the lesions, they were gradually incised. Bacteriological, mycological, and virological testing confirmed a herpetic rash without superinfection or superimposed dermatosis.

**Figure 1:**
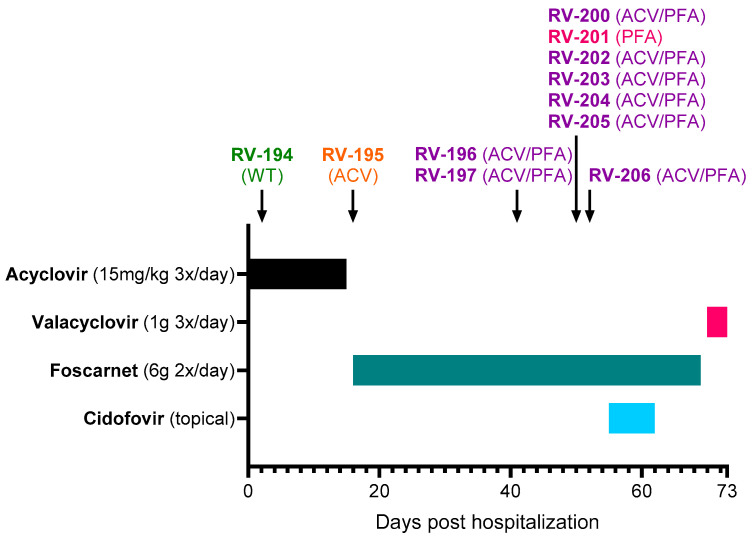
Overview of the clinical course and treatment of a primary HSV-2 infection in a patient with medullary aplasia. The day of hospitalization was considered Day 0. The isolation dates of the clinical samples are indicated by the arrows and the resistance profiles are shown in parenthesis. Abbreviations: ACV, acyclovir; PFA, foscarnet; WT, wild type.

The rash was initially very extensive, but the lesions on the face, trunk, and roots of the limbs quickly regressed. However, new lesions continued to appear despite acyclovir treatment, and treatment was switched to foscarnet (6 g 2×/day) on Day +16 (Figure 1). At Day +18, inflammation was observed around the herpetic lesions, and vancomycin was initiated to prevent superinfections. Foscarnet treatment did not allow a favorable evolution, with the persistence and appearance of new skin lesions. The new lesions were predominantly on the palm and fingertips of the left hand, left foot, and mouth and subsequently on the right palm, sole, and pinna. Skin improvement only occurred after initiation of topical cidofovir treatment (Day +55 to Day +62). Cidofovir (1% cream) was applied to all skin lesions except the left hallux and mucosal lesions. After the start of this treatment, no new sites were affected. Healing of the mucocutaneous lesions coincided with a gradual reduction in pain. The patient was sampled on Day +70, a few days before her discharge, and the samples were all positive by culture. The favorable evolution of the lesions made it possible to stop foscarnet on Day +70 and start valacyclovir (1 g 3×/day), which was maintained for an undetermined period after her discharge on Day +73.

### 3.2. Resistance Monitoring of Clinical HSV-2 Samples

Eleven clinical samples were collected from different lesion sites between Day +2 and Day +52 (Figure 1 and Table 1). The first samples were received by the RegaVir platform (www.regavir.org) for genotyping and susceptibility testing at Day +45. The first isolate (RV-194), recovered from a buccal lesion at Day +2, had a wild-type genotype and phenotype. The other 10 isolates showed resistance to acyclovir (RV-195), foscarnet (RV-201), or both drugs (RV-196, RV-197, RV-200, and RV-202 to RV-206).

TK mutations were found in samples RV-195 (R221H) and RV-205 (C deletion at position 246–249) (Table 1). All samples collected after initiation of foscarnet treatment (Day +16) carried mutations in the DP gene. An unexpectedly high level of heterogeneity was found between the different lesion sites since none of the 11 samples had the same composition of mutations. In total, Sanger sequencing detected 10 different DP mutations among the samples, i.e., A606V, A724V, S725G, S729N, I731F, Q732R, M789T, V842M, R847C, and R964H.

### 3.3. Sanger Sequencing of Plaque-Purified Viral Clones

Multiple virus clones were plaque-purified from each clinical sample and genotyped by Sanger sequencing to study the observed viral heterogeneity in more detail (Table 2). Clones harboring mutations that were not detected by initial Sanger sequencing were isolated from six of the clinical samples. In total, 22 distinct types of virus clones were isolated, namely a wild-type virus, one TK mutant, eighteen DP mutants, and two TK/DP double mutant virus strains.

Clones with identical genotypes within each respective sample were obtained from the first three samples, i.e., RV-194 (wild-type), RV-195 (TK R221H mutant), and RV-196 (DP T934A mutant). Surprisingly, none of the RV-196-derived clones harbored the DP A606V mutation, which was detected as a mixed population with the wild-type virus in the original sample by Sanger sequencing. All other samples proved to be mixed populations, either of wild-type and mutant viruses (RV-197 and RV-200) or of 2 to 8 different mutant viruses (RV-201, RV-202, RV-203, RV-204, RV-205, and RV-206). Two TK/DP double-mutant virus strains were isolated from RV-205, which both bore a C deletion in the TK gene at nucleotide position 246–249 in combination with either the DP Q732R or R847C mutation.

The highest level of heterogeneity was observed in samples RV-197 and RV-200, from which eight and five different types of virus strains were isolated, respectively. From sample RV-197, wild-type clones and clones harboring the DP K533E, G617S, C625R, R628C, or S725G mutations were isolated in addition to DP R964H (14/42 clones) and DP V842M (3/42 clones) mutant viruses, which were also detected by initial Sanger sequencing of the clinical sample. From RV-200, wild-type clones (1/14) and clones bearing the DP R628C (3/14), F923L (7/14), I950L (1/14) or R964H (2/14) mutation were isolated. Two types of virus clones were isolated from the remaining clinical samples (RV-201 to RV-206).

### 3.4. Drug Susceptibility of Plaque-Purified Virus Clones

**Figure 2:**
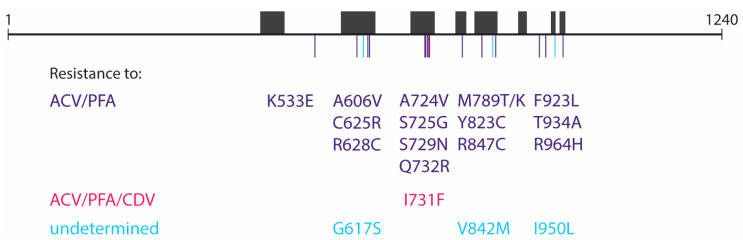
Schematic overview of DNA polymerase mutations detected during primary HSV-2 infection in a patient with medullary aplasia. Conserved domains within the DNA polymerase protein are shown by the black boxes. Abbreviations: ACV, acyclovir; PFA, foscarnet; CDV, cidofovir.

The TK R221H mutant clone was resistant to the nucleoside analogs acyclovir (51.4-fold) and ganciclovir (4-fold) but remained susceptible to foscarnet, adefovir, and cidofovir (Table 3). The two TK/DP double mutants (TK C del at nucleotides 246–249 + DP Q732R and TK C del at nucleotides 246–249 + DP R847C) were both highly resistant to acyclovir and ganciclovir, due to the presence of the TK frameshift mutation. In addition, both double mutants showed resistance to foscarnet and adefovir. The DP mutations K533E, A606V, C625R, R628C, A724V, S725G, S729N, Q732R, M789T/K, Y823C, R847C, F923L, T934A, and R964H were found to confer resistance to acyclovir and foscarnet, and the I731F mutation to acyclovir, foscarnet, and cidofovir (Table 3), as has been reported in a previous publication [12]. The effects of the DP G617S, V842M, and I950L mutations could not be verified by CPE reduction assays as they failed to grow in cell culture. A schematic overview of the 19 DP mutations detected in the virus clones and their associated resistance profile can be found in Figure 2.

### 3.5. Deep Sequencing of the DP Gene of Clinical HSV-2 Samples Shows High Genetic Diversity

As more drug-resistant mutations were identified among the plaque-purified viruses than in the original clinical samples, targeted deep sequencing of the DP gene was performed on the clinical samples (Table 4). RV-194 and RV-195 had a wild-type DP gene, which was in agreement with initial Sanger sequencing. In contrast, the other nine samples bore two or more mutations detected at frequencies varying from 1.1% to 95.4%. Additional DP mutations, not detected by Sanger sequencing of the original clinical samples or in the plaque-purified virus clones, were detected in six out of eleven clinical samples, being RV-196 (Y823C), RV-197 (A606V, G631D, A840T, I950L), RV-200 (D616G, S725G, A840T), RV-201 (A724V), RV-202 (G607D), and RV-206 (R628C). In agreement with the diversity of plaque-purified clones, the highest variability was observed in RV-197 (eleven mutations) and RV-200 (seven mutations).

### 3.6. Passaging of Clinical Samples in Cell Culture Decreases Viral Diversity

The percentages of variants identified by deep sequencing did not always correlate with the proportion of mutant clones found by plaque purification. To examine whether some of the DP mutants had altered viral fitness, the original clinical samples were cultured for five passages on HEL fibroblasts, after which deep sequencing of the DP gene was performed (Table 4). Following five passages, RV-194 and RV-195 retained their wild-type DP genotype. In the other cultured samples, only one or two mutations remained present. In most cases, the remaining mutants already had the highest frequency in the non-cultured clinical samples, i.e., RV-200 (F923L from 32.1% before passaging to 99.8% at passage 5), RV-201 (I731F, 94% to 99.8%), RV-202 (Q732R, 95.4% to 83.8%), RV-203 (S725G, 71.7% to 71.4%), RV-205 (Q732R, 54.7% to 88.5%), and RV-206 (S729N, 64.8% to 99.4%). However, in RV-196, RV-197, and RV-204, one of the minor variants became the predominant variant after five passages in cell culture. In sample RV-196, the T934A mutant variant increased in frequency from 12.6% to 97.5%, outgrowing the A606V (41.6% to undetectable levels) and Y823C (35.2% to 1.5%) mutants. In RV-204, the A606V mutant (85% to undetectable levels) was also outgrown, in this case by the Y823C mutant (10.3% to 98.6%). In RV-197, the R964H variant (27.1% to 98%) outgrew all other variants, including the G631D and V842M mutant, which were originally detected at a 31% and 42.9% frequency, respectively. In isolate RV-203, the frequencies of the A724V (27.6% to 28.3%) and S725G (71.7% to 71.4%) variants did not seem to be affected by the repetitive passaging in cell culture.

### 3.7. Growth Kinetics of HSV-2 Mutant Variants

**Figure 3:**
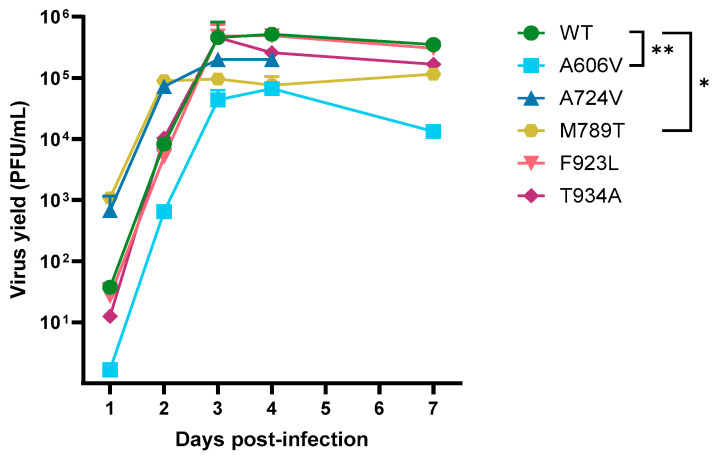
Growth kinetics of HSV-2 wild-type and DNA polymerase mutant strains. HEL fibroblasts were infected with the different viruses at a concentration of 100 PFU/well in 24-well plates. Plates were frozen at 1-, 2-, 3-, 4-, and 7 days post infection, and virus yields were determined upon thawing. Data are means and SD for two replicates. *, *p* < 0.05; **, *p* < 0.01.

The growth kinetics of the DP A606V, A724V, M789T, F923L and T934A mutants and a patient-derived wild-type strain were evaluated in absence of antiviral drug pressure (Figure 3). The A724V, F923L and T934A mutants did not show a significant change in replication capacities compared to the wild-type virus. However, the viral growth of the A606V mutant was significantly decreased to that of the wild type at all timepoints (*p* < 0.01). Interestingly, the M789T mutant virus showed a significant increase in replication at days 1 and 2 post infection (*p* < 0.05), but a decreased replication capacity at days 3, 4, and 7 postinfection (*p* < 0.05).

### 3.8. DNA Polymerase Mutants Show Altered Fitness in Comparison to Wild Type

**Figure 4:**
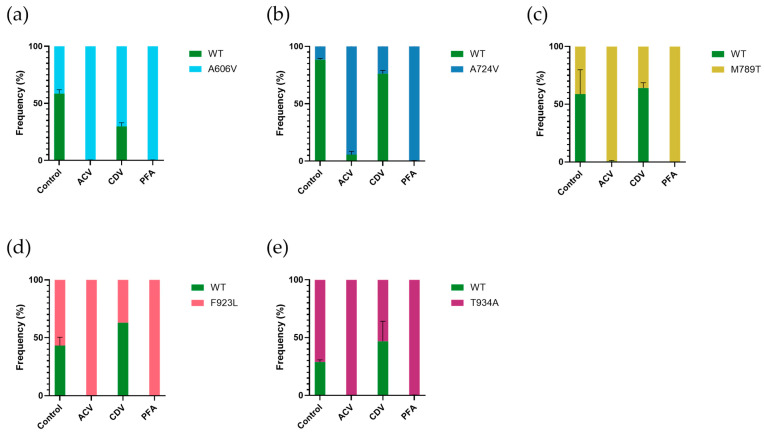
Competition assays of the wild-type virus with various HSV-2 DNA polymerase mutants. HEL cells were co-infected with clinical wild-type (WT) and mutant virus stocks mixed in a 50:50 ratio. Growth competition was evaluated without drug pressure (control) and under pressure of acyclovir (ACV, 1 µg/mL), cidofovir (CDV, 1 µg/mL), and foscarnet (PFA, 100 µg/mL). The frequencies of each virus were quantified 7 days post infection by deep sequencing. Data shown are averages from two experiments with standard deviations: (**a**) competition assays of the wild-type virus versus the DP A606V mutant virus; (**b**) competition assays of the wild-type virus versus the DP A724V mutant virus; (**c**) competition assays of the wild-type virus versus the DP M789T mutant virus; (**d**) competition assays of the wild-type virus versus the DP F923L mutant virus; and (**e**) competition assays of the wild-type virus versus the DP T934A mutant virus.

The replication capacity of the DP A606V, A724V, M789T, F923L, and T934A mutants relative to the wild-type virus were evaluated by competition assays in absence of antiviral drugs or under antiviral pressure (Figure 4 and Table S2). When competition experiments were conducted in absence of antiviral pressure, the A606V, M789T, and F923L mutants were detected at frequencies equal to the wild-type virus (Figure 4a,c,d). The A724V mutant had a lower frequency (11.6%, Figure 4b), whereas the T934A mutant (71.2%, Figure 4e) had a higher frequency than the wild type.

All mutant viruses outgrew the wild-type virus under acyclovir pressure (frequencies ranging from 94.4% for A724V to 99.7% for the T934A mutant) and under foscarnet pressure (frequencies > 99%), in agreement with their resistance profile. No significant change in frequency was observed under cidofovir pressure, except for the DP A606V mutant, which had a higher frequency following cidofovir pressure compared to the untreated control (70.2% versus 41.4%, *p* = 0.03).

### 3.9. Mutant-Versus-Mutant Competition Experiments

**Figure 5:**
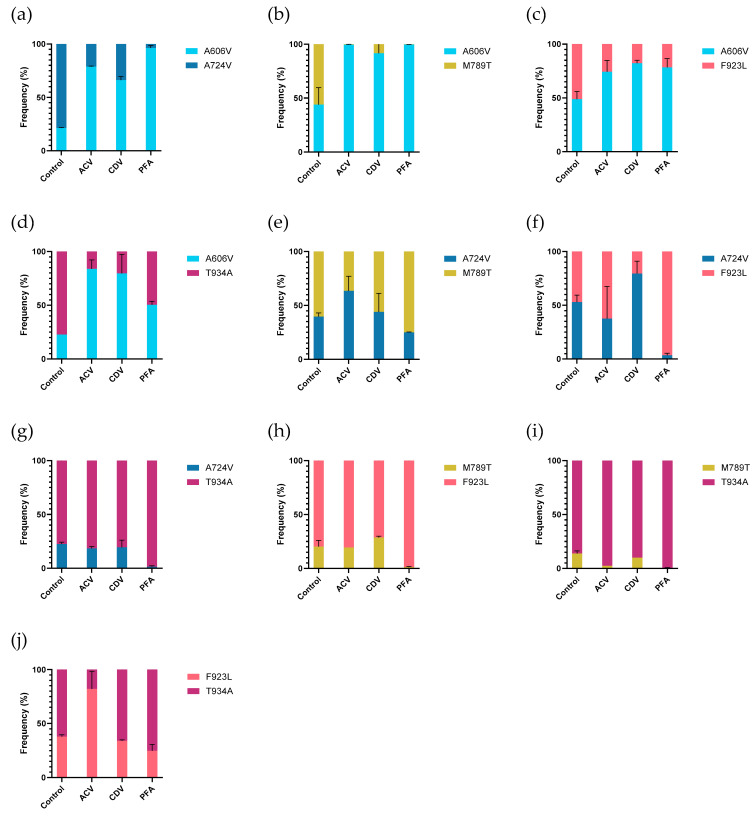
HSV-2 mutant-versus-mutant competition assays in presence or absence of antiviral pressure. Viral growth competition was evaluated without drug pressure (control) and under pressure of acyclovir (ACV, 1 µg/mL), cidofovir (CDV, 1 µg/mL), and foscarnet (PFA, 100 µg/mL). The frequency of each virus was quantified 7 days post infection by deep sequencing. Data shown are averages from two experiments with standard deviations. Competition assay of mutant viruses: (**a**) A606V and A724V; (**b**) A606V and M789T; (**c**) A606V and F923L; (**d**) A606V and T934A; (**e**) A724V and M789T; (**f**) A724V and F923L; (**g**) A724V and T934A; (**h**) M789T and F923L; (**i**) M789T and T934A; and (**j**) F923L and T934A.

Next, the relative replication capacities of the five selected mutant viruses were tested in mutant-versus-mutant competition assays (Figure 5 and Table S3). Without drug pressure, most mutants had equal frequencies 7 days post infection when incubated in the absence of drug pressure, although a few differences were observed. The DP A606V mutant had a lower frequency in competition assays with the A724V and T934A mutants (21.4% and 22.1%, respectively). The T934A mutant revealed higher frequencies when co-infected with the A724V or DP M789T mutants, which were detected at 22.5% and 13.8%, respectively. The DP M789T mutant also had a lower frequency in competition assays with the DP F923L mutant (20.2%).

The A606V mutant was detected at higher frequencies in all mutant-versus-mutant competition assays (vs. A724V, M789T, F923L, and T934A) under acyclovir pressure in comparison to the untreated control experiments, suggesting a fitness gain under acyclovir pressure. The F923L mutant showed a higher frequency in competition with T934A (37.9% in untreated vs. 82.1% in acyclovir treated). In the other competition assays, no obvious change in frequencies were observed under acyclovir pressure.

Under cidofovir pressure, the A606V mutant was detected at high frequencies in competition with all mutant viruses, ranging from 66.2% in competition with A724V to 91.6% in competition with mutant M789T. The A724V mutant had a higher frequency in competition with the F923L mutant, in comparison to the untreated control condition (53% vs. 79.5%). For all other mutant-versus-mutant conditions, no differences in frequency between the cidofovir treated and untreated conditions were observed.

The A606V mutant again revealed higher frequencies in all competition experiments under foscarnet pressure, compared to assays performed in absence of antiviral pressure. The A724V and M789T mutants were outgrown by all other mutants, in agreement with the lowest level of foscarnet resistance observed for this mutant (2.7-fold and 3.2-fold, respectively). No significant differences in frequencies were observed in the A724V vs. M789T (39.7% vs. 44%) and F923L vs. T934A (37.9% vs. 34.1%) experiments under foscarnet pressure, compared to untreated control experiments.

## 4. Discussion

Large DNA viruses like HSV-2 are generally considered genetically more stable than RNA viruses due to the proofreading capacity of the viral DP, allowing the excision of mismatched nucleotides during replication [13]. However, in this study we report a case of primary HSV-2 infection in an immunocompromised patient that rapidly progressed to a multidrug-resistant infection, characterized by heterogeneous viral populations comprising over 20 distinct drug-resistant variants and viral compartmentalization.

Although HSV genomes (i.e., HSV-1 and HSV-2) can remain static for decades in immunocompetent individuals [14,15,16], prolonged replication when the immune system is impaired or, in the presence of selective pressure, such as antiviral exposure, can ultimately have a significant impact on intra-host diversity. Our findings align with previous work by us and others that revealed heterogeneous HSV populations in immunocompromised patients [11,17,18,19]. High levels of intra-host viral variation offer a rapid mechanism of evolution, allowing minor variants to quickly emerge as dominant upon the introduction of selective pressure [20]. The high diversity of drug-resistant variants described in our patient highlights the adaptive capacity of HSV-2 in the face of antiviral therapy, complicating both first- and second-line therapies.

The limited availability of HSV antivirals necessitates the responsible use of antivirals, preferably guided by a drug resistance profile analysis rather than an empirical approach. Notably, in our patient, viral samples collected the same day from different lesion sites all exhibited distinct compositions of viral variants. This degree of viral compartmentalization complicates drug resistance diagnostics, as the site of sample collection may impact the mutations that will be detected. Furthermore, resistance genotyping is almost exclusively performed by Sanger sequencing, which cannot detect minor drug-resistant variants with frequencies below 20% [5]. Implementation of deep sequencing approaches, such as the targeted Illumina sequencing that we performed retrospectively, would improve the detection of low-frequency variants and has already been adopted by some reference laboratories [18,21].

High levels of viral heterogeneity further complicate the management of HSV-2 infections as standard antiviral regimens may fail to control heterogeneous infections, leading to the persistence of drug-resistant strains. In our patient, lesions only improved after initiation of a combined regimen of foscarnet and topical cidofovir. Similarly, multiple reports found combination therapies, such as concurrent use of acyclovir and foscarnet, to be effective in treating refractory HSV infections [5]. These findings suggest that tailored combination therapy may be required to effectively treat HSV-2 infections involving multiple strains with varying drug susceptibilities.

In recent years, a novel class of antivirals targeting the helicase-primase complex have proceeded clinical trials, including amenamevir and pritelivir. Amenamevir was recently approved in Japan for the treatment of recurrent HSV infections [22], and a Phase 3 clinical trial evaluating pritelivir for the treatment of acyclovir-resistant HSV infections in immunocompromised subjects (NCT03073967) is ongoing. Pritelivir is already available through an expanded access program and has demonstrated efficacy in treating recurrent HSV-2 infections in several case reports [23,24,25]. Given their distinct molecular target, both amenamevir and pritelivir could potentially have been effective in treating the multidrug-resistant infection observed in our patient.

Antiviral resistance is a delicate equilibrium of the virus between evading the antiviral action and maintaining its replication capacity. Resistance mutations often incur a fitness cost in the absence of antiviral pressure [26]. In this study we revealed variations in the fitness cost associated with different DP mutations. This was evident when the clinical samples were passaged in cell culture, resulting in the loss of several mutant variants over time. Interestingly, in three out of nine samples with mixed DP populations, a minor variant outgrew the major variant during passaging. This suggests differential fitness dynamics of the strains in cell culture compared to the host environment in the presence of antiviral pressure. An example is the A606V mutant, which was the major variant in the clinical samples RV-196 and RV-204 but was outgrown by other mutants (T934A and Y823C, respectively) upon passaging in cell culture, in the absence of antiviral pressure. This was further supported by the reduced replication capacity of the A606V mutant found in the growth curve kinetics assays. However, the A606V mutant outgrew the wild-type virus and all mutant variants in competition assays performed under antiviral pressure. The dual-infection competition assays revealed that drug pressure significantly enhanced the fitness of resistant mutants, allowing them to outgrow the wild-type virus under antiviral pressure with frequencies reaching almost 100%. This fitness advantage was not observed when competition assays were performed under exposure to drugs to which the mutant viruses remained susceptible. Our results underscore the complex interplay between resistance mutations, viral replication capacity, and drug selection pressure.

The accumulating evidence on heterogeneity in clinical HSV infections supports the use of competition assays over the traditional comparison of growth curves in monocultures. A limitation of the current study was that the viral DNA ratio in the inoculum was not quantified by deep sequencing. Though by adding a 50:50 ratio of the infectious virus doses, we expect that the DNA frequency of both viruses in the inoculum was approximately 50%. There might be variations in the viral DNA-to-infectious particle ratios among different strains, which could have influenced initial frequencies, complicating interpretations of the fitness data. Future studies should include deep sequencing of the viral inoculum to improve the interpretation of results.

In summary, this study addresses the adaptability of HSV-2 in an immune suppressed host and under antiviral pressure, revealing substantial heterogeneity and compartmentalization. These characteristics complicate both the diagnosis of drug resistance and the treatment of HSV-2 infections. Our findings underscore the necessity of developing tailored therapeutic approaches that integrate resistance profiling and combination therapy to address the rising threat of multi-drug resistant HSV infections. Moreover, ongoing surveillance and a deeper understanding of resistance mechanisms are critical to improve outcomes in immunocompromised individuals facing persistent HSV-2 infections.

## Figures and Tables

**Table 1:** Phenotype and genotype of clinical HSV-2 samples obtained from different body sites during primary infection.

Isolate	Day of Isolation ^a^	Sample Origin	Mutation in ^b^		Fold-Resistance ^c^				
			Thymidine Kinase	DNA Polymerase	ACV	GCV	PFA	ADV	CDV
RV-194	+2	Buccal swab	-	-	1.1	1.3	0.9	1.2	1.1
RV-195	+16	Skin swab	R221H	-	**66.7**	**5.8**	0.4	NP	0.5
RV-196	+41	Skin swab	-	A606V ^d^	**6.7**	1.2	**5.4**	NP	1.4
RV-197	+41	Skin swab	-	V842M ^d^, R964H ^d^	**5.4**	1.3	**5.1**	NP	1.7
RV-200	+50	Right hand	-	R964H ^d^	**3.6**	1.2	**3.4**	**3.2**	1.0
RV-201	+50	Left foot	-	I731F	1.3	0.9	**3.8**	1.6	1.1
RV-202	+50	Sole left foot	-	Q732R	**6.7**	1.8	**3.7**	**3.2**	0.7
RV-203	+50	Left hallux	-	A724V ^d^, S725G	**5.9**	1.4	**4.0**	**4.9**	0.9
RV-204	+50	Palm left hand	-	A606V	**3.2**	0.8	**2.6**	**2.6**	0.7
RV-205	+50	Left index	C del nts 246–249	Q732R ^d^, R847C ^d^	**≥526**	**839**	**3.6**	**6.3**	0.7
RV-206	+52	Buccal swab	-	S729N, M789T ^d^	**2.9**	1.1	**3.8**	**6.8**	1.6

^a^ The day of hospitalization was defined as Day 0; ^b^ mutations detected by Sanger sequencing of the clinical sample; ^c^ the mean 50% effective concentration (EC_50_) of at least two independent experiments was used to calculate the fold resistance (EC_50_ clinical sample/EC_50_ wild-type G strain); ^d^ mutation was found as a mix; NP: assay not performed. Abbreviations: ACV, acyclovir; GCV, ganciclovir; PFA, foscarnet; ADV, adefovir; CDV, cidofovir.

**Table 2:** Genotype of HSV-2 virus clones plaque-purified from clinical samples.

Clinical Sample (Genotype ^a^)	No. of Clones (%)	Mutations in Viral	
		Thymidine Kinase	DNA Polymerase
**RV-194** (wild type)	**2/2 (100%)**	-	-
**RV-195** (TK R221H)	**5/5 (100%)**	**R221H**	-
**RV-196** (DP A606V ^b^)	**21/21 (100%)**	-	**T934A**
**RV-197** (DP V842M ^b^, R964H ^b^)	7/42 (16.7%)	-	-
	2/42 (4.8%)	-	K533E
	1/42 (2.4%)	-	G617S
	5/42 (11.9%)	-	C625R
	6/42 (14.3%)	-	R628C
	4/42 (9.5%)	-	S725G
	3/42 (7.1%)	-	V842M
	**14/42 (33.3%)**	**-**	**R964H**
**RV-200** (DP R964H ^b^)	1/14 (7.1%)	-	-
	3/14 (21.4%)	-	R628C
	**7/14 (50%)**	-	**F923L**
	1/14 (7.1%)	-	I950L
	2/14 (14.3%)	-	R964H
**RV-201** (DP I731F)	1/10 (10%)	-	C625R
	**9 (90%)**	-	**I731F**
**RV-202** (DP Q732R)	**10 (90.9%)**	-	**Q732R**
	1 (9.1%)	-	M789K
**RV-203** (DP A724V ^b^, S725G)	3/18 (16.7%)	-	A724V
	**15/18 (83.3%)**	-	**S725G**
**RV-204** (DP A606V)	**4/8 (50%)**	-	**A606V**
	**4/8 (50%)**	-	**Y823C**
**RV-205** (DP Q732R ^b^, R847C ^b^)	**9/10 (90%)**	**C del nt 246–249**	**Q732R**
	1/10 (10%)	C del nt 246–249	R847C
**RV-206** (DP S729N, M789T ^b^)	4/19 (21.1%)	-	S729N
	**15/19 (78.9%)**	-	**M789T**

^a^ Genotype that was detected by Sanger sequencing of the clinical sample. ^b^ Detected as mixed population in the clinical sample by Sanger sequencing. The predominant type of virus clone isolated from each sample is marked in bold.

**Table 3:** Resistance profiles of several plaque-purified HSV-2 virus clones harboring mutations in the thymidine kinase and/or DNA polymerase genes.

Virus Clone(s)	Mutation in		Fold-Resistance ^b^				
	Thymidine Kinase	DNA Polymerase	ACV	GCV	PFA	ADV	CDV
RV-195 clone 4	R221H	-	**51.4**	**4.0**	0.7	1.0	0.4
RV-205 clone 4	C del nt 246–249	Q732R	**≥1122**	**559**	**5.4**	**3.2**	1.2
RV-205 clone 21	C del nt 246–249	R847C	**≥225**	**827**	**5.0**	**11.8**	1.2
RV-197 clones 31 and 32 ^a^	-	K533E	**3.8**	1.0	**4.2**	**3.2**	1.4
RV-204 clones 1–4 ^a^	-	A606V	**6.2**	0.8	**6.2**	**10**	1.7
RV-197 clones 4, 33, and 39 ^a^	-	C625R	**3.3**	1.4	**4.4**	**6.1**	1.4
RV-200 clones 3 and 9 ^a^	-	R628C	**2.0**	0.6	**2.4**	**2.1**	1.2
RV-203 clones 19, 23, and 25 ^a^	-	A724V	**3.6**	0.5	**2.7**	**2.4**	1.0
RV-203 clones 1–6 ^a^	-	S725G	**2.7**	0.5	**4.0**	**3.6**	0.7
RV-206 clone 6 ^a^	-	S729N	**6.9**	1.2	**6.9**	**9.7**	1.6
RV-201 clones 1, 2, and 4 ^a^	-	I731F	**2.9**	0.8	**6.0**	**3.6**	**2.3**
RV-202 clones 3 and 5 ^a^	-	Q732R	**3.8**	0.8	**4.1**	**4.3**	1.8
RV-206 clones 1–4 and 6 ^a^	-	M789T	**3.3**	0.7	**3.2**	**4.0**	1.4
RV-202 clone 4 ^a^	-	M789K	**3.4**	0.6	**3.3**	**3.1**	1.6
RV-204 clones 5, 19, 21, and 23 ^a^	-	Y823C	**3.4**	0.6	**2.0**	**2.6**	1.3
RV-200 clones 1, 2, and 6 ^a^	-	F923L	**7.5**	0.8	**4.7**	**4.5**	1.4
RV-196 clones 1–6 ^a^	-	T934A	**5.0**	0.8	**5.4**	**4.8**	1.1
RV-197 clone 2 ^a^	-	R964H	**4.9**	0.4	**4.8**	**7.6**	1.0

^a^ Original data can be found in Andrei et al. 2018 [12]; ^b^ EC_50_ of mutant virus clone/EC_50_ of a wild-type clone derived from patient isolate RV-194. Fold resistance levels ≥ 2 are marked in bold. Abbreviations: ACV, acyclovir; GCV, ganciclovir; PFA, foscarnet; ADV, adefovir; CDV, cidofovir.

**Table 4:** Frequency of DNA polymerase mutations detected in clinical HSV-2 samples before and after passaging in cell culture.

Clinical Sample	Mutation	Frequency (%) Detected by NGS		% Clones with Mutation
		Passage 0	Passage 5	
RV-194	-	-	-	100%
RV-195	-	-	-	100%
RV-196	A606V ^a^	41.6%	-	0%
	Y823C	35.2%	1.5%	0%
	T934A	12.6%	97.5%	100%
RV-197	K533E	3.4%	-	4.8%
	A606V	6.0%	-	0%
	G617S	1.7%	-	2.4%
	C625R	8.0%	-	11.9%
	R628C	1.8%	-	14.3%
	G631D	31%	-	0%
	S725G	1.1%	-	9.5%
	A840T	1.5%	-	0%
	V842M ^a^	42.9%	-	7.1%
	I950L	1.4%	-	0%
	R964H ^a^	27.1%	98%	33.3%
RV-200	D616G	3.0%	-	0%
	R628C	16.2%	-	21.4%
	S725G	2.4%	-	0%
	A840T	11.9%	-	0%
	F923L	32.1%	99.8%	50%
	I950L	12.6%	-	7.1%
	R964H ^a^	26.0%	-	14.3%
RV-201	A724V	3.5%	-	0%
	I731F ^a^	94%	99.8%	90%
RV-202	G607D	2.1%	-	0%
	Q732R ^a^	95.4%	83.8%	90.9%
	M789K	8.7%	11.5%	9.1%
RV-203	A724V ^a^	27.6%	28.3%	16.7%
	S725G ^a^	71.7%	71.4%	83.3%
RV-204	A606V ^a^	85%	-	50%
	Y823C	10.3%	98.6%	50%
RV-205	Q732R ^a^	54.7%	88.5%	90%
	R847C ^a^	30.4%	-	10%
RV-206	R628H	3.1%	-	0%
	S729N ^a^	64.8%	99.4%	21.1%
	M789T ^a^	57.1%	-	78.9%

^a^ Mutation was detected by Sanger sequencing of the original clinical sample prior to passaging.

## Data Availability

The original contributions presented in the study are included in the article/Supplementary Materials; deep sequencing data have been submitted to the GenBank SRA database under BioProject PRJNA1219479. Further inquiries can be directed to the corresponding author.

## Supplementary Materials

The following supporting information can be downloaded at: https://www.mdpi.com/article/10.3390/v17070962/s1; Table S1: Primers used for Sanger sequencing; Table S2: Frequencies and statistics of HSV-2 mutants in competition experiments with wild-type virus; Table S3: Frequencies and statistics of HSV-2 mutant-versus-mutant competition experiments.

## Abbreviations

The following abbreviations are used in this manuscript:

ACV	Acyclovir
ADV	Adefovir
CCID_50_	50% cell culture infective dose
CDV	Cidofovir
CPE	Cytopathic effect
DMEM	Dulbecco’s modified Eagle’s medium
DP	DNA polymerase
FBS	Fetal bovine serum
GCV	Ganciclovir
HEL	Human embryonic lung
HSV	Herpes simplex virus
HSV-1	Herpes simplex virus 1
HSV-2	Herpes simplex virus 2
PFA	Foscarnet
PFU	Plaque forming unit
TK	Thymidine kinase
