# Supplementary material for: Primary HSV-2 Infection in an Immunocompromised Patient Reveals High Diversity of Drug-Resistance Mutations in the Viral DNA Polymerase
Source: Viruses. 2025 Jul 9;17(7):962. doi: 10.3390/v17070962 (PMC12298504; doi:10.3390/v17070962)
Supplement: Supplementary file 1 [file viruses-17-00962-s001.zip › viruses-3732787-supplementary.pdf]

Table S1. Primers used for Sanger Sequencing

Gene	Amplicon	Forward primer (5′-3′)	Reverse primer (5′-3′)
UL23 (thymidine kinase)	A	GTCAGCAGCGTTCCACAAATCCT	CCCGATATGAGGAGCCAAAACG
	B	GTAATGACCAGCGCCCAGATAAC	CGTCCAAGACCCAGGCAAAAATG
	C	GTCCGCCATTCGCCGTGTC	GTTCTTTTATTGCCGTCATCGCC
UL30 (DNA polymerase)	A	CATCCCACCCCGAGCTGTTG	GACCCAGAAGCGTGATGACGG
	B	ACCCCACCGTCACCGTCTTC	CCAGGTTGTCCGCCGTGC
	C	CCACCCGGTTTATCCTGGACAAC	ACCATCCCGTTCACCTTGATCTTG
	D	TCTACAAGGTCCCGCTCGACG	CCTCGTCGTCGTCCTTATCCTCG
	E	GTTTCGGGGCCTCGACAAGG	CGAGCGGATCTGCTTTCGCA
	F	ACCGGGACTACCTGGAGATCGAG	CTTGGCGATGAGCAGCAGCT
	G	TGGGCGACAAGATGGCGAG	CCTTGATGGACGGGACCTGC
	GH	GGTCGACCTGCTGTTTTACG	CGGTGATCTTGGCGTTATTT
	H	GCAGGTCCCGTCCATCAAGG	CGTGGTCAGACGCAACGCAG

Table S2. Frequencies and statistics of HSV-2 mutants in competition experiments with wild-type virus.

**Genotype of mutant** treatment condition	**Frequency (%)**	**p value**[a]	**Fitness interpretation**[b]
**DP A606V**			
Without drug	41.4 ± 3.4		
ACV (1 µg/mL)	99.6 ± 0.1	**0.04**	↑
CDV (1 µg/mL)	70.2 ± 3.2	**0.03**	↑
PFA (100 µg/mL)	99.6 ± 0.1	**0.04**	↑
**DP A724V**			
Without drug	11.6 ± 0.9		
ACV (1 µg/mL)	94.4 ± 2.7	**0.03**	↑
CDV (1 µg/mL)	23.8 ± 2.9	0.18	=
PFA (100 µg/mL)	99.6 ± 0.1	**0.008**	↑
**DP M789T**			
Without drug	41.2 ± 21.1		
ACV (1 µg/mL)	99.4 ± 0.6	0.26	=
CDV (1 µg/mL)	36.0 ± 4.7	0.97	=
PFA (100 µg/mL)	99.9 ± 0.1	0.26	=
**DP F923L**			
Without drug	56.9 ± 7.3		
ACV (1 µg/mL)	99.8 ± 0.06	0.11	=
CDV (1 µg/mL)	37.0		
PFA (100 µg/mL)	99.9 ± 0.002	0.11	=
**DP T934A**			
Without drug	71.2 ± 1.9		
ACV (1 µg/mL)	99.7 ± 0.1	**0.049**	↑
CDV (1 µg/mL)	53.3 ± 17.4	0.6	=
PFA (100 µg/mL)	99.8 ± 0.2	**0.049**	↑

[a] Significant p-values ($p < 0.05$) are marked bold. [b] Relative fitness of mutant relative to wild-type. Abbreviations: ACV, acyclovir; CDV, cidofovir; DP, viral DNA polymerase; PFA, foscarnet.

Table S3. Frequencies and statistics of HSV-2 mutant-versus-mutant competition experiments.

Genotype of competing viruses treatment condition	Frequency (%)[a]	p value[b]	Fitness interpretation
**DP A606V *vs* DP A724V**			
Without drug	21.4 ± 0.5		
ACV (1 µg/mL)	78.8 ± 0.6	**0.0002**	A606V > A724V
CDV (1 µg/mL)	66.2 ± 3.3	0.06	=
PFA (100 µg/mL)	96.3 ± 2.5	**0.03**	A606V > A724V
**DP A606V *vs* DP M789T**			
Without drug	44.0 ± 15.7		
ACV (1 µg/mL)	99.4 ± 0.5	0.2	=
CDV (1 µg/mL)	91.6 ± 10.5	0.1	=
PFA (100 µg/mL)	99.7 ± 0.05	0.2	=
**DP A606V *vs* DP F923L**			
Without drug	48.9 ± 7.0		
ACV (1 µg/mL)	74.3 ± 10.6	0.3	=
CDV (1 µg/mL)	82.2 ± 2.8	**0.03**	A606V > F923L
PFA (100 µg/mL)	78.4 ± 8.1	0.1	=
**DP A606V *vs* DP T934A**			
Without drug	22.1 ± 1.1		
ACV (1 µg/mL)	83.9 ± 8.3	0.1	=
CDV (1 µg/mL)	79.5 ± 17.8	0.2	=
PFA (100 µg/mL)	50.4 ± 3.2	0.09	=
**DP A724V *vs* DP M789T**			
Without drug	39.7 ± 3.3		
ACV (1 µg/mL)	63.5 ± 13.6	0.4	=
CDV (1 µg/mL)	44.0 ± 17.2	0.97	=
PFA (100 µg/mL)	24.9 ± 0.5	0.2	=
**DP A724V *vs* DP F923L**			
Without drug	53.0 ± 6.4		
ACV (1 µg/mL)	37.5 ± 30.1	0.9	=
CDV (1 µg/mL)	79.5 ± 11.5	0.2	=
PFA (100 µg/mL)	3.6 ± 1.9	0.1	=
**DP A724V *vs* DP T934A**			
Without drug	22.5 ± 1.7		
ACV (1 µg/mL)	18.5 ± 1.7	0.3	=
CDV (1 µg/mL)	19.4 ± 6.7	0.9	=
PFA (100 µg/mL)	1.4 ± 0.9	**0.009**	T934A > A724V
**DP M789T *vs* DP F923L**			
Without drug	20.2 ± 5.9		
ACV (1 µg/mL)	19.5		
CDV (1 µg/mL)	28.7 ± 1.22	0.4	=
PFA (100 µg/mL)	1.3 ± 0.6	0.2	=
**DP M789T *vs* DP T934A**			
Without drug	13.8 ± 2.5		
ACV (1 µg/mL)	2.4		
CDV (1 µg/mL)	9.9		
PFA (100 µg/mL)	0.4 ± 0.4	0.08	=
**DP F923L *vs* DP T934A**			
Without drug	37.9 ± 1.9		
ACV (1 µg/mL)	82.1 ± 16.4	0.3	=
CDV (1 µg/mL)	34.1 ± 0.9	0.4	=
PFA (100 µg/mL)	24.8 ± 6.0	0.3	=

[a] Frequencies of mutant 1 are indicated. [b] Significant p-values ($p < 0.05$) are marked bold. Abbreviations: ACV, acyclovir; CDV, cidofovir; DP, viral DNA polymerase; PFA, foscarnet.
